# *Astilbe Chinensis* ethanol extract suppresses inflammation in macrophages via NF-κB pathway

**DOI:** 10.1186/s12906-020-03073-5

**Published:** 2020-10-07

**Authors:** Tae-Young Gil, Bo-Ram Jin, Chul-Hee Hong, Jong Hyuk Park, Hyo-Jin An

**Affiliations:** 1 Department of Pharmacology, College of Korean Medicine, Sangji University, 83, Sangjidae-gil, Wonju-si, Gangwon-do 26339 Republic of Korea; 2 Department of Korean Medicine Ophthalmology & Otolaryngology & Dermatology, College of Korean Medicine, Sangji University, 83, Sangjidae-gil, Wonju-si, Gangwon-do 26339 Republic of Korea; 3 Hallym University, Chuncheon, Republic of Korea

**Keywords:** *Astilbe chinensis*, Macrophages, NF-κB pathway, Inflammation, In vitro, Ex vivo

## Abstract

**Background:**

Macrophages play a crucial role in inflammation. *Astilbe chinensis* is one of perennial herbs belonging to the genus *Astilbe.* Plants in the genus have been used for pain, headaches, arthralgia, and chronic bronchitis. However, the effect of *A.chinensis* on inflammation remains unclear. To study the anti-inflammatory action of *A.chinensis* ethanol extract (ACE), we investigated the effect of ACE on the production of pro-inflammatory mediators and cytokines in macrophages.

**Methods:**

We evaluated the effectiveness of ACE in lipopolysaccharide (LPS)-stimulated RAW 264.7 macrophages and thioglycollate (TG)-elicited peritoneal macrophages from male C57BL/6 mice. We measured the levels of pro-inflammatory mediators and cytokines, and examined the anti-inflammatory actions of ACE on nuclear factor κB (NF-κB) pathway in the macrophages. Western blot analysis and immunofluorescence microscopy were used to determine protein level and translocation, respectively.

**Results:**

ACE suppressed the output of nitric oxide (NO), prostaglandin E2 (PGE_2_), and pro-inflammatory cytokines in stimulated macrophages via inhibiting the expression of inducible nitric oxide synthase (iNOS) and cyclooxygenase-2 (COX-2) proteins. ACE suppressed mRNA expression of pro-inflammatory cytokines such as interleukin (IL)-6 and tumor necrosis factor-alpha (TNF-α). We examined the efficacies of ACE on NF-κB activation by measuring the expressions including IκB kinase (IKK), inhibitor of κB (IκB), and nuclear p65 proteins. In addition, the inhibition of NF-κB p65’s translocation was determined with immunofluorescence assay.

**Conclusion:**

Our findings manifested that ACE inhibited LPS or TG-induced inflammation by blocking the NF-κB signaling pathway in macrophages. It indicated that ACE is a potential therapeutic mean for inflammation and related diseases.

## Background

The inflammatory affair is a successive and well-modulated mechanism against the various stimulations and initial step in the defense system. Target cells like macrophages can be induced by physical, microbial, chemical, and immune logical reactions producing inflammatory responses [1]. However, prolonged or chronic inflammation causes various chronic diseases including cancers, diabetes mellitus, and metabolic syndromes. Macrophages are crucial in directing the host immune response against infection as well as in many pathophysiological processes associated with inflammation. They are strong secretory cells releasing a set of pro-inflammatory mediators [2]. Nitric oxide (NO), prostaglandin E_2_ (PGE_2_), tumor necrosis factor-alpha (TNF-α), and interleukins (ILs) are pro-inflammatory mediators and cytokines which promote inflammatory responses in macrophages [3]. On this note, the expressive amounts of pro-inflammatory mediators and cytokines are considered as indices of the degree of inflammation.

Lipopolysaccharide (LPS)-induced inflammation in macrophages is widely used in inflammatory studies. LPS is the dominant element of endotoxins derived from Gram-negative bacterial outer membrane [4]. LPS induces inflammatory responses and facilitates the yield of pro-inflammatory mediators and cytokines which is done by binding to Toll-like receptor 4 (TLR4) [5]. The inflammatory mediators and cytokines can be modulated via the activation of transcription factors, such as nuclear factor-κB (NF-κB), mediated by LPS-induced signaling cascade [6]. NF-κB what is assembled with p65 and p50 subunits which regulate multiple transcriptional factors including inducible enzymes such as inducible nitric oxide synthase (iNOS), cyclooxygenase-2 (COX-2), and pro-inflammatory cytokines [7]. NF-κB is isolated as an inactive complex bound to inhibitor of κB (IκB) in the cytoplasm. The IκB proteins are swiftly phosphorylated in the matter of pro-inflammatory stimuli and they are eventually degraded via the proteosomal pathway which is involved in the formation of IκB kinase (IKK) complexes [8].

*Astilbe chinensis* (*A.chinensis*) (Maxim.) Franch. & Sav. (ID: kew-2,656,728) belongs to the family *Saxifragaceae*, and usually grows in moist fields and mountains [9]. Plants in the genus *Astilbe*, including *A.chinensis*, have been known to nurse chronic bronchitis, arthralgia, inflammation, pain, and headache. The dried roots of *A.chinensis* and its chemical constituents have been used not only as antipyretic and analgesic remedies but also for the treatment of bronchitis [10]. In addition, there are studies on various effects such as the anti-obesity [11], platelet modulating [12], and α-glucosidase inhibitory activities of *A.chinensis* [13]. There are bioactive compounds from *A.chinensis*, such as astilbic acid, astilbin, and bergenin (Table 1). The anti-inflammatory activity of astilbic acid has been shown in bone marrow-derived mast cells [17] and in allergic asthma [14]. Because inflammation is deeply involved in innate immunity, this study would figure out the effect in the other immune cells. However, the efficacies of ACE on inflammation have not been investigated in macrophages. Hence, this study would investigate the anti-inflammatory effectiveness of ACE in murine RAW 264.7 and primary peritoneal macrophages from male C57BL/6 mice. Since we would like to study the effect of ACE as much as possible related to human biology, we applied it on peritoneal macrophages, the most often used as a model system in macrophages-related functional studies.

**Table 1:** Active compound of *Astilbe chinensis*

Active compound	PubChem CID	Reference
Astilbic acid	12,016,586	(Yuk, Lee, Kwon, Cai, Jang, Oh, Lee and Ahn 2011 [14])
Astilbin	119,258	(Chen et al. 2018 [15])
Bergenin	66,065	(Chen and Nie 1988 [16])

## Methods

### Chemicals and reagents

Dulbecco’s modified Eagle’s medium (DMEM), fetal bovine serum (FBS), penicillin, and streptomycin were got from Life Technologies Inc. (Grand Island, NY, USA). LPS (*Escherichia coli*, serotype 055:B5), 3-(4,5-dimethylthiazol-2-yl)-2,5-diphenyltetrazolium bromide (MTT), N6-(1-Iminoethyl) lysine (NIL), NS-398, and Griess reagent were bought from Sigma Chemical Co. (St. Louis, MO, USA). Dimethyl sulfoxide (DMSO) was purchased from Junsei Chemical Co. Ltd. (Tokyo, Japan). Primary antibodies including iNOS, COX-2, NF-κB p65, p-IκB-α, IκB-α, and β-actin monocolonal antibodies were bought from Santa Cruz Biotechnology (Santa Cruz, CA, USA). p-IKK-α/β, IKK-α/β, and PARP antibodies were got from Cell Signaling Technology (Danvers, MA, USA). Horseradish peroxidase-conjugated secondary antibodies and normal goat serum were got from Jackson Immuno Research laboratories, Inc. (West Grove, PA, USA). SYBR green master mix was obtained from Applied Biosystem (Foster, CA, USA). IL-6, TNF-α, and glyceraldehyde-3-phosphate dehydrogenase (GAPDH) oligonucleotide primers were got from Bioneer (Daejeon, Republic of Korea). The enzyme-linked immunosorbent assay (ELISA) kits for IL-6, TNF-α, and PGE_2_ were obtained from R&D Systems (Minneapolis, MN, USA). Mounting medium with 4,6-diamidino-2-phenylindole (DAPI) was got from Vector Laboratories, Inc. (CA,USA). Alexa Flour 488 goat anti-rabbit IgG H&L was obtained from Invitrogen Corp (Carlsbad, CA, USA).

### Preparation of ethanol extract of Astilbe chinensis (ACE)

ACE was a gift from Institute of Natural Cosmetic Industry for Namwon (Namwon, Jeollabuk-do, Republic of Korea). The dried and powdered rhizome of plant material was extracted thrice by maceration with 95% ethanol. The extract was evaporated in vacuo at 40 °C, was filtered, and freeze-dried *in vaccum*. The freeze-dried sample was dissolved in DMSO at a final concentration of 50 mg/mL for the bio assays.

### Cell culture and sample treatment

The RAW 264.7 macrophages cell line was purchased from Korea Cell Line Bank (KCLB, Seoul, Republic of Korea). This cell line was cultured in DMEM supplemented with 10% FBS, penicillin (100 U/mL), and 1% streptomycin (100 μg/mL) in 37 °C and 5% CO_2_ incubator. ACE was dissolved in DMSO and the cells were treated with 12.5, 25, or 50 μg/mL ACE. The cells (1 × 10^5^ cells/mL) were stimulated with 1 μg/mL of LPS for the indicated time prior to treatment with ACE for 1 h.

### Experimental animals and sample treatment

Male C57BL/6 mice (*n* = 4; 8 weeks old) were got from Daehan Biolink Co. (Daejeon, Republic of Korea). All animals were housed in accordance with the guidelines for the care and use of laboratory animals. The guidelines were adopted and promulgated by Sangji University according to the requirements demonstrated by the National Institutes of Health. All the experimental protocols were approved based on the Institutional Animal Care and Use Committee (IACUC) of Sangji University before the beginning of the study (IACUC Animal approval protocol No.2018–3). The mice were housed in a cage and fed standard laboratory chow in the animal room with 12 h dark/light cycles and constant condition; 20 ± 5 °C temperature, 40–60% humidity) for a week. We got the primary peritoneal cells from the mice using the protocol described formerly [18, 19]. We used 4% brewer thioglycollate (TG, Difco, Laboratories, Detroit, MI, USA) to isolate the peritoneal macrophages under the inflammation. Each mouse was intraperitoneally administered 3 mL of 4% TG for 4 days. Prior collection of the peritoneal primary macrophages from the mice, we sacrificed them in accordance with the IACUC animal approval. Every effort was made to minimize animal suffering. Animals were fasted for 12 h and euthanized by cervical dislocation. To harvest the primary cells from mice, we performed peritoneal lavage with 8 ml of Hank’s balanced salt solution (HBSS, Gibco BRL, Grand Island, NY, USA) containing 10 U/mL heparin. The cells were distributed in DMEM in 24-well tissue culture plates (3 × 10^5^ cells/well) and were incubated for 3 h at 37 °C under an atmosphere of 5% CO_2._ They were washed three times with HBSS to remove non-adherent cells, and equilibrated with DMEM supplemented with 10% FBS before sample treatment. ACE was dissolved in DMSO and the cells were treated with 12.5, 25, or 50 μg/mL ACE. The cells were stimulated with 20 U/mL IFN-γ (BD Pharmingen™, BD Biosciences, USA) for 6 h, and with 1 μg/mL of LPS for the indicated time prior to treatment with ACE for 1 h.

### NO production assay

NO content was measured indirectly by assaying the culture supernatant for nitrite with Griess reagent (1% sulfanilamide in 5% phosphoric acid, 1% α-naphthylamide in H_2_O). NO production from the macrophages was in form of NO_2_ in the culture medium. The cells were distributed in DMEM IN 24-well culture plates (1 × 10^5^ cells/mL) and were incubated for 48 h. Cell culture media (50 μL) was mixed with 50 μL of Griess reagent in a 96-well plate and incubated at room temperature for 15 min followed by the measurement of the absorbance at 540 nm using an automatic microplate reader (Titertek Multiskan). Values are presented as mean ± S.D. of three independent experiments.

### PGE_2_ assay

The macrophages (1 × 10^5^ cells/mL) were treated with ACE for 1 h prior to stimulation with LPS (1 μg/mL). After 24 h, the level of PGE_2_ in the culture media was measured using PGE_2_ enzyme immune assay kit (R&D Systems, Minneapolis, MN, USA). The experiments were performed in triplicate. Values are presented as mean ± S.D. of three independent experiments.

### Cytokine assays

RAW 264.7 macrophages (1 × 10^5^ cells/mL) were pre-treated with ACE for 1 h prior to the addition of LPS. The culture media were collected about 24 h post-treatment with ACE and stored at − 80 °C. The levels of IL-6 and TNF-α were measured with EIA kits according to the manufacturer’s instructions. Values are presented as mean ± S.D. of three independent experiments.

### Quantitative real-time PCR analysis (qRT-PCR)

RAW 264.7 macrophages (1 × 10^5^ cells/mL) were homogenized, and total RNA was isolated using an easy-BLUE™ total RNA extraction kit (iNtRON Biotechnology Inc., Gyeonggi-do, South Korea). cDNA was obtained using isolated total RNA (1 μg), d(T)16 primer, and avian myeloblastosis virus reverse transcriptase (AMV-RT). Relative gene expression was quantified with real-time PCR (Real Time PCR System 7500, Applied Biosystems, CA, USA) with SYBR green PCR mast mix (Applied Biosystems, CA, USA). The gene Ct values of IL-6 and TNF-α were normalized with the gene express 2.0 program (Applied Biosystems, CA, USA) to the Ct values of GAPDH. Values are presented as mean ± S.D. of three independent experiments.

### Nuclear extraction

RAW264.7 macrophages were plated in 60-mm dishes (1 × 10^5^ cells/mL) and pre-treated with ACE for 1 h prior to the addition of LPS. After 30 min, the cells washed three times with PBS, scraped into 1 mL of cold PBS, and pelleted by centrifugation. Cell pellets were resuspended in hypotonic buffer (10 mM HEPES, pH 7.9, 1.5 mM MgCl_2_, 10 mM KCl, 0.2 mM PMSF, 0.5 mM DTT, 10 μg/mL aprotinin). Then, cells incubated on ice for 15 min. Next, cells were lysed by adding 0.1% Nonidet P-40 and vortexed vigorously for 30 min. Nuclei were pelleted by centrifugation at 12,000×g for 2 min at 4 °C and resuspended in high salt buffer (20 mM HEPES, pH 7.9, 25% glycerol, 400 mM KCl, 1.5 mM MgCl_2_, 0.2 mM EDTA, 0.5 mM DTT, 1 mM NaF, 1 mM sodium orthovandate).

### Western blot analysis

Protein extracts were isolated from the cell lines (1 × 10^5^ cells/mL) using the protein lysis buffer Pro-prep™ (Intron biotechnology Inc., Gyeonggi-do, South Korea). Protein samples were separated on an 8–12% sodium dodecyl sulphate-polyacryl–amide gel. After electrophoresis, proteins were transferred to polyvinylidenedifluoride membranes. The membranes were blocked with 2.5–5% skim milk for 30 min and incubated overnight with specific primary antibodies in Tris-buffered saline (TBS) containing 0.1% Tween20 at 4 °C. Primary antibody was removed by washing the membrane three times in TBS-T buffer, and incubated for 2 h with horseradish peroxidase-conjugated secondary antibody (1:2500) at 25 °C. After washing thrice in TBS-T, immuno-detection bands were reacted with ECL solution (Absignal, Seoul, Republic of Korea) and captured on X-ray film (Agfa, Belgium). Values are presented as mean ± S.D. of three independent experiments.

### Immunofluorescence assay

Cells (1 × 10^5^ cells/mL) were cultured directly on the camber slide (Lab-Tek II chamber slide #154526, 4 well) for 24 h to detect NF-κB/p65 localization. After stimulation with LPS in the presence or absence of ACE, the cells were fixed with 100% methanol for 30 min at room temperature and blocked with 10% normal goat serum. The cells were incubated overnight with specific primary antibodies in 10% blocking solution. After washing the primary antibodies with 0.3% Triton X in PBS for 30 min, Alexa fluor 488 goat anti-rabbit IgG was applied for 1 h. Cells were mounted with mounting medium containing 4′, 6-diamidino–2–phenylindole (DAPI) and observed under a fluorescence microscope.

### Statistical analysis

Results are expressed as the mean ± S.D. of triplicate experiments. Statistically significant differences were determined using ANOVA and Dunnett’s post hoc test, and *p-values* > 0.05 indicated statistical significance.

## Results

### Effects of ACE on the production of inflammatory mediators in RAW 264.7 macrophages

**Fig. 1:**
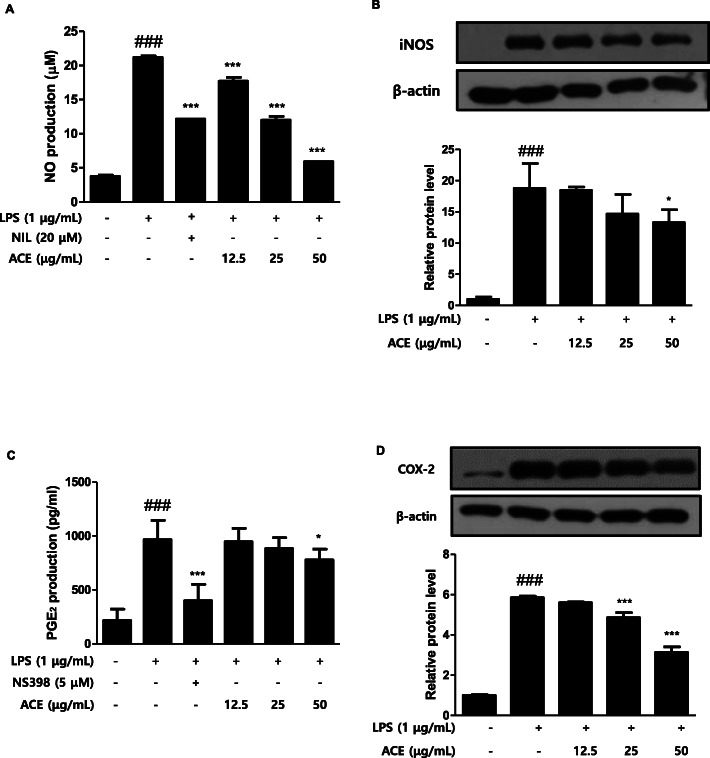
Effects of ACE on the levels of inflammatory mediators in RAW 264.7 macrophages. Cells were treated with 12.5, 25, or 50 μg/mL of ACE for 1 h prior to the addition of LPS (1 μg/mL), and the cells were incubated for 24 h and 48 h, respectively. **a** NO and (**c**) PGE_2_ level were figured out with Griess reagent and the EIA kit, respectively. NIL (20 μM) or NS398 (5 μM) was used as positive control. The protein level of (**b**) iNOS and (**d**) COX-2 were determined by western blot analysis with specific antibodies. Densitometric analysis was performed with Image J software (version 1.50i). Values are presented as mean ± S.D. of three independent experiments. ^###^*p* < 0.001 when compared with control; ^*^p < 0.05, ^***^p < 0.001 when compared with LPS-induced group. Significant differences among treated groups were determined by ANOVA and Dunnett’s post hoc test

NO is a major mediator in the inflammatory system [20] and hence, controlling its production has an important meaning in the investigation on an anti-inflammation. The level of NO was figured out with Griess reaction test. Whereby, the nitrite ion in the sample was measured by comparing its absorbance with the prepared standard [20]. We confirmed the inhibitory effectiveness of ACE on NO production in murine macrophage cell line (1 × 10^5^ cells/mL) (Fig. 1a). There was about four-fold increase in NO production in LPS-stimulated group compared to the control group. However, ACE suppressed NO production in a dose-dependent manner in the cells. The inhibitory action of 25 μg/mL ACE on NO production was similar to that of the positive control, NIL (20 μM). When macrophages are exposed to LPS, the expression of iNOS ultimately results in the overproduction of NO [21]. iNOS is an NO synthase, which indicates that the suppression of iNOS expression directly correlates with NO production in inflammatory responses [22]. We examined the level of expression of iNOS protein in RAW 264.7 macrophages (1 × 10^5^ cells/mL) with western blot assay to determine if the previous inhibitory effect was connected to the modulation of the expression of iNOS. Compared to the control group, LPS (1 μg/mL) caused an increase in the expression of iNOS protein. Pre-treatment with ACE (50 μg/mL) reduced the expression of iNOS significantly (Fig. 1b).

PGE_2_ is commonly considered a pro-inflammatory signaling molecule [23] and thus, we examined its output in murine RAW 264.7 cell line (1 × 10^5^ cells/mL) (Fig. 1c). The cellular exposure to LPS remarkably stimulated the yield of PGE_2_ compared with control group. However, treatment with ACE inhibited the making of PGE_2_ in a dose-dependent manner in macrophages. The highest concentration of ACE (50 μg/mL) showed a significant down-regulation on PGE_2_ production. COX-2, one of the inducible enzymes associated with excessive production of PGE_2_, acts as pro-inflammatory mediators in inflammatory state [24]. Hence, we assessed the effectiveness of ACE on the expression of COX-2 protein in RAW 264.7 macrophages (1 × 10^5^ cells/mL) with western blot assay (Fig. 1d). The LPS-stimulated group showed higher expression of COX-2 protein than the LPS-untreated group. However, the expressive level of COX-2 protein was down-regulated in a dose-dependent manner by ACE. Pre-treatment of RAW 264.7 macrophages with 25 and 50 μg/mL ACE showed significant suppression.

### Effects of ACE on LPS-induced pro-inflammatory cytokines in RAW264.7 macrophages

**Fig. 2:**
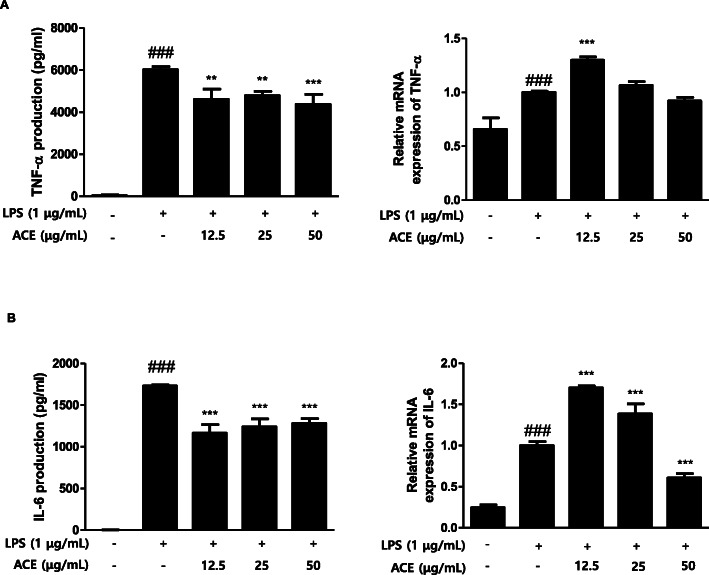
Effects of ACE on LPS-induced pro-inflammatory cytokines in RAW 264.7 macrophages. Cells were treated with 12.5, 25, or 50 μg/mL of ACE for 1 h prior to the addition of LPS (1 μg/mL). The cells were further incubated for 24 h, and cytokine production was measured with EIA kits. After 6 h of incubation with LPS, mRNA expression of TNF-α and IL-6 were determined with quantitative real-time PCR. The data shown represent mean ± S.D. of three independent experiments. ^###^*p* < 0.001 when compared with control; ^**^p < 0.01, ^***^p < 0.001 when compared with LPS-stimulated group. Significant differences between treated groups were determined by ANOVA and Dunnett’s post hoc test

Cytokines are crucial local protein mediators got involved in various biological processes for instance cell growth and activation, inflammation, immunity, and differentiation. They have pivotal role in autoimmune diseases [25]. Pro-inflammatory cytokines participate in the prolonging of chronic inflammation [20]. Therefore, the regulation of these cytokines could be a budding strategy in the control of inflammation or related diseases. To investigate the effect of ACE on the yield of pro-inflammatory cytokines for example TNF-α and IL-6 in LPS-stimulated macrophages (1 × 10^5^ cells/mL), we carried out qRT-PCR to measure the mRNA expression of the cytokines (Fig. 2a), and ELISA for cytokine production (Fig. 2b). With regards to mRNA expression, the cell pre-treated with ACE showed attenuated expression level in a dose-dependent manner even though the group with the lowest concentration of ACE (12.5 μg/mL) had a higher mRNA expression of the cytokines than the untreated LPS-induced group (Fig. 2a). Furthermore, TNF-α and IL-6 production levels in the LPS-stimulated group markedly increased comparing the control group. Yet, treatment with 12.5, 25, and 50 μg/mL of ACE markedly diminished the production of cytokines (Fig. 2b).

### Effects of ACE on LPS-induced NF-κB pathway in RAW 264.7 macrophages

**Fig. 3:**
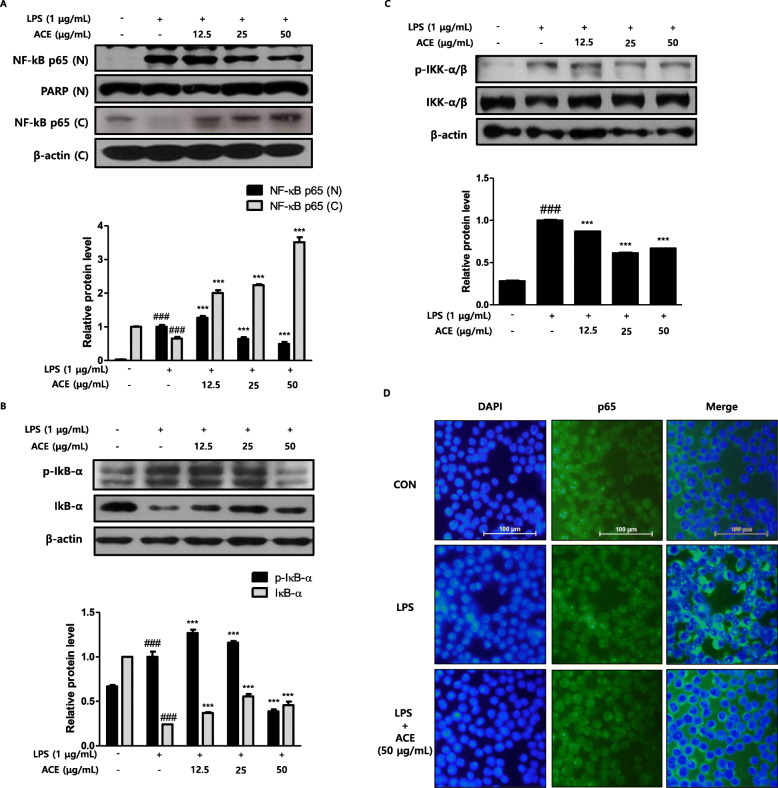
Effects of ACE on LPS-induced NFκB pathway in RAW 264.7 macrophages. **a** Cells were treated with 12.5, 25, or 50 μg/mL of ACE for 1 h prior to the addition of LPS (1 μg/mL). LPS stimulation took time on NF-κB p65 for 30 min. Nuclear (N) and cytosolic (C) extracts were isolated and adjusted for the detection of p65 with specific antibodies. After incubation with LPS for 15 min (**b**) and 5 min (**c**), total proteins were prepared and western blot assay was performed with specific antibodies. PARP and β-actin were shown as internal controls. Densitometric analysis was performed with Image J software (version 1.50i). Data are presented as mean ± S.D. of three independent experiments. ^###^*p* < 0.001 when compared with control; ^***^p < 0.001 when compared with LPS-induced group. Significant differences between treated groups were determined by ANOVA and Dunnett’s post hoc test. **d** Cells were pre-treated with ACE for 1 h prior to the addition of LPS for 30 min. The nuclear translocation of NF-κB p65 was visualized by immunofluorescence. The nuclei were counterstained with DAPI (blue). The stained cells were visualized with a fluorescence microscope at 400X magnification

As controlling the transcription of various genes, NF-κB has an pivotal role in the development of acute and chronic inflammatory diseases [26]. We evaluated the effectiveness of ACE on NF-κB activation in macrophages (1 × 10^5^ cells/mL) through measurement the protein expression and translocation of NF-κB p65 (Fig. 3). As shown in the figures, NF-κB p65 translocation to the nucleus was inhibited by ACE in a dose-dependent manner. Western blot assay with cytosolic and nuclear fractions expressed increased amount of NF-κB p65 in the nucleus of the LPS-stimulated group for 30 min (Fig. 3a). As shown in Fig. 3d, the inhibitory action of ACE on translocation of NF-κB p65 was assessed in each group.

NF-κB is segregated in the cytoplasm which is done by binding to inhibitory proteins like IκBs [27], and thus, we experimented on the phosphorylation of IκB in LPS-stimulated RAW 264.7 macrophages. As shown in Fig. 3b, ACE markedly suppressed IκBα phosphorylation in a dose dependent manner and restored IκBα degradation.

The IKK complex incorporates two catalytic subunits, IKKα and IKKβ. It regulates the activation of NF-κB transcription factors, which plays a crucial activity in inflammation [28]. Figure 3c showed the protein expression of IKKα/β in the groups. Only LPS-stimulated group showed activated IKK comparing to the control group. At 12.5, 25, and 50 μg/mL, ACE blocked the activation of IKK significantly.

### Effects of AxCE on LPS-induced production of inflammatory mediators in peritoneal macrophages

**Fig. 4:**
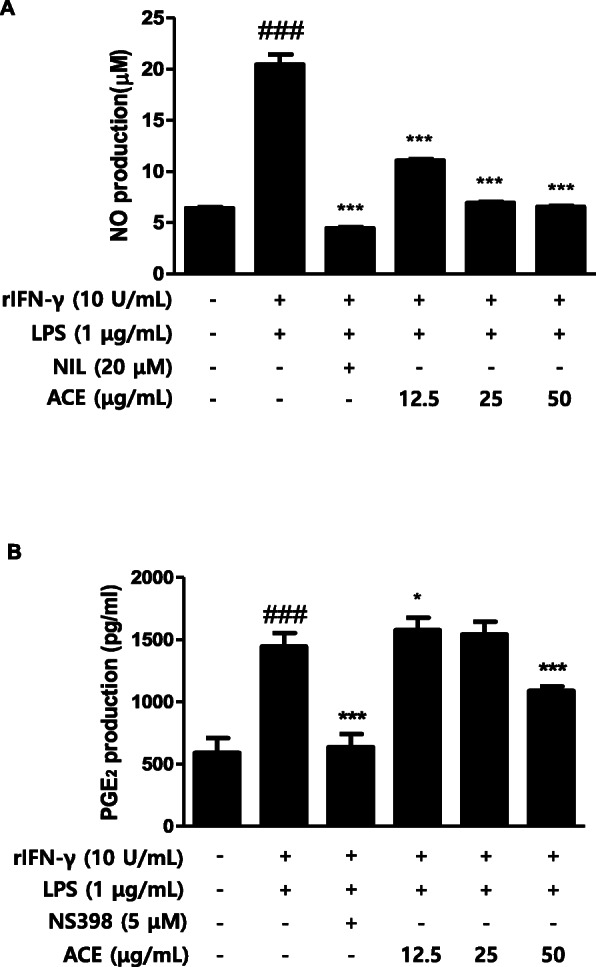
Effects of ACE production of inflammatory mediators in stimulated primary peritoneal macrophages. Thioglycollate (3%), rIFN-γ (10 U/mL), and LPS (1 μg/mL)-stimulated primary peritoneal macrophages were incubated for 48 h. **a** NO and (**b**) PGE_2_ level were measured with Griess reagent and the EIA kit, respectively. NIL (20 μM) or NS398 (5 μM) was used as positive control. Values are presented as mean ± S.D. of three independent experiments. ^###^*p* < 0.001 when compared with control; ^*^p < 0.05, ^***^p < 0.001 when compared with LPS-induced group. Significant differences between treated groups were determined by ANOVA and Dunnett’s post hoc test

As crucial cellular effectors to nonspecific host defense, macrophages emit different inflammatory mediators in the immune system [2]. Primary peritoneal macrophages were isolated from the mice under TG treatment. It took 3 days to isolate the stimulated primary cells after which they were incubated for 24 h for stabilization. After the pre-treatment with ACE and positive control for 1 h, the cells were exposed to rIFNγ and LPS for 6 h and 48 h, respectively, to induce inflammatory responses. We confirmed the suppressive effects of ACE on NO and PGE_2_ production in peritoneal macrophages (3 × 10^5^ cells/mL) (Fig. 4). As shown in Fig. 4a, NO production was increased by about four folds in rIFNγ- and LPS-stimulated groups comparing to the control group. However, the production was down-regulated by ACE in a dose-dependent manner. The groups treated with 25 and 50 μg/mL of ACE showed similar levels of production to the positive control group, NIL (20 μM). In Fig. 4b, the level of PGE_2_ increased significantly in rIFNγ- and LPS-stimulated groups against the control group. The production level of the positive control, NS398 (5 μM), in the stimulated groups was the same as that in the non-stimulated group. In addition, the highest concentration of ACE (50 μg/mL) showed a significant inhibition of PGE_2_ production.

## Discussion

Inflammation is a host protective response against numerous alien pathogens or tissue injury. It occurs in organisms not only to remove harmful stimuli but also to evoke the curing and repairing affairs for damaged tissues [29, 30]. However, prolonged inflammation causes health problems [31]. Hence, this study examined the innate immune system as related to inflammation.

In traditional medicine, *A.chinensis* is known for its anti-inflammatory, anti-cancer, anti-obesity and hepato-protective effects [11, 13]. Foregoing studies showed the anti-inflammatory efficacy of astilbic acid from *A.chinensis* on mast cells [17], and its protective effect on Ultraviolet B-injured keratinocytes [9] suggesting possibility as anti-inflammatory means. Also, there are other constituents for the effects including astilbin for LPS-induced macrophages [32] and bergenin for synovial inflammation [33]. In this context of the effective elements, we assumed there is synergistically anti-inflammatory effect. Therefore, we figured out the efficacies of ACE on LPS or TG-treated macrophages.

Macrophages are the primary cells in the immune system which originated as blood monocytes [3]. They are important cellular effectors in nonspecific host defense, producing an array of inflammatory mediators, bioactive lipids, and hydrolytic enzymes which are involved in tissue injury [2]. In the present study, the levels of inflammatory mediators such as NO and PGE_2_ were determined in murine RAW 264.7 macrophages and primary peritoneal macrophages. Macrophages in the peritoneum under non-elicited state are usually not enough for experimental studies. Sterile eliciting agents such as Brewer’s TG broth or proteose peptone can be used in the peritoneal cavity to rise the yield of macrophages [34]. Either cell line or peritoneal macrophage was stimulated with LPS, which is a strong activator of TLR4 signal. LPS triggers the most influential microbial initiator in the inflammatory responses for instance septic shock, microbial violation, or fever [35]. LPS evokes inflammatory responses and binding complex in macrophages through Toll-like family receptors and the co-receptor, CD14 [36]. Binding with receptors, especially TLR4, causes phosphorylation and induces nuclear factor kappa-light-chain-enhancer of activated B cell (NF-κB) inflammatory signaling pathway [37]. In this study, we figured out that ACE exhibited anti-inflammatory effect in LPS-treated macrophages through NF-κB signaling pathway.

Macrophages participate in immune responses which are accompanied by increased level of inflammatory mediators [38]. We compared the action of ACE on the yield of inflammatory mediators in homogenous population of peritoneal macrophages and in murine cell line. ACE suppressed the production of both NO (Fig. 1a, Fig. 4 a) and PGE_2_ (Fig. 1 c, Fig. 4 b)_._ Although NO has a critical character in various body functions, its exceeding production in macrophages causes inflammation, cytotoxicity, or autoimmune disorders [39]. The efficacy of ACE on the expression level of iNOS, one of the key enzymes promoting the production of NO from arginine as response to different inflammatory stimuli [40], was examined (Fig. 1b). Another important mediator, PGE_2_, is made by the inducible enzyme COX-2 and it is related to the events constituting diverse chronic inflammatory diseases [41]. COX-2 produces PGs which causes inflammatory symptoms [42]. The restrained expression of COX-2 by ACE is shown in Fig. 1d. We found that ACE decreased the protein expression of pro-inflammatory mediators, NO and PGE_2,_ as well as their inducible enzymes, iNOS and COX-2. LPS-stimulated macrophages and their production are considered as inflammatory mediators. They induce other pro-inflammatory cytokines namely TNF-α and IL-6 which have been linked to the process in the chronic inflammatory diseases [43]. Both TNF-α and IL-6 are the central mediators of sepsis, which is uncontrolled inflammatory response which can result in multi-organ failure and even demise [44]. In this study, the actions of ACE on the levels of the pro-inflammatory cytokines were examined (Fig. 2).

**Fig. 5:**
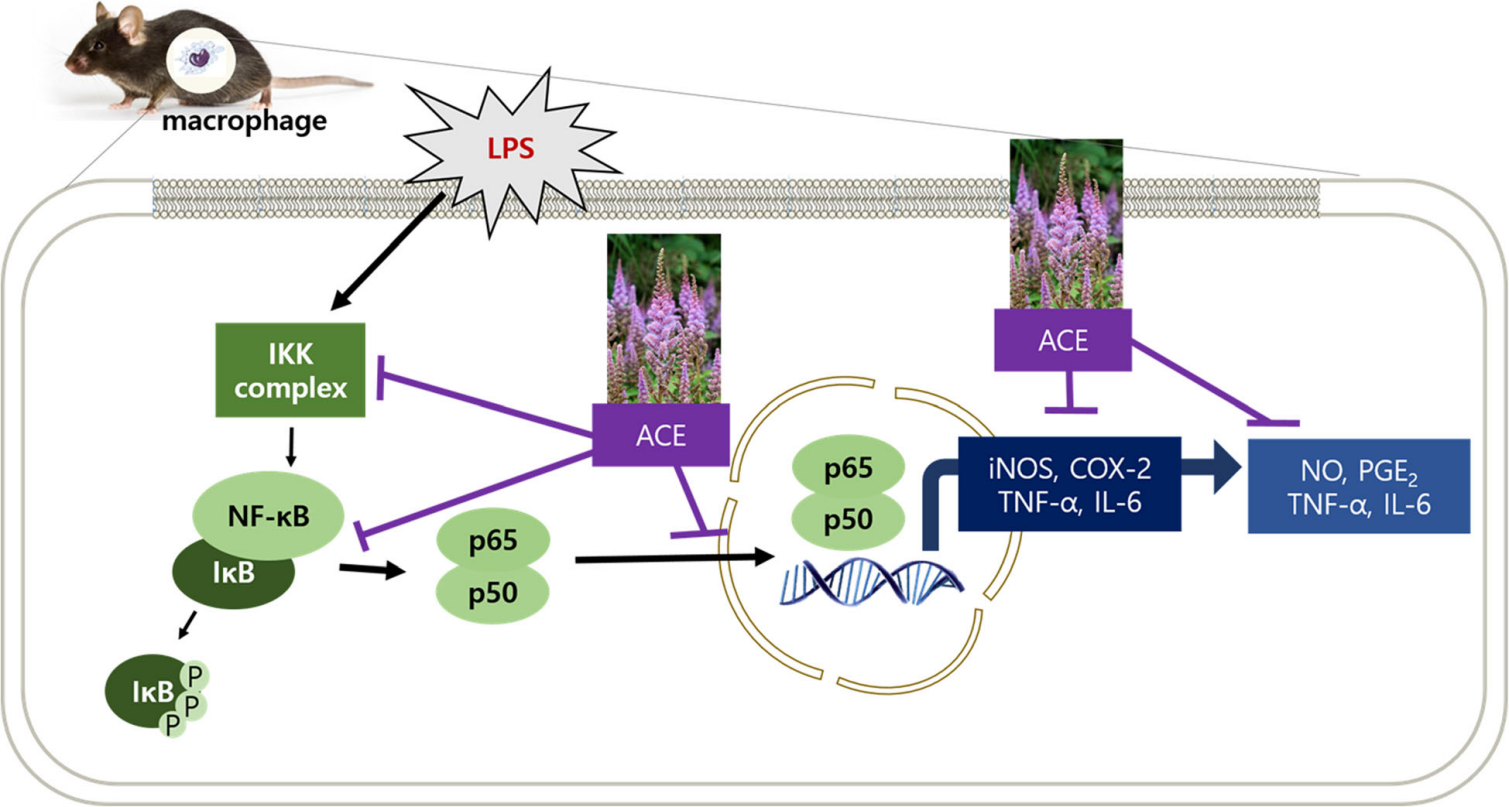
The mechanism of ACE on inflammation in murine macrophages

The LPS-leading signaling cascade induces the activation of NF-κB signaling pathways in either myeloid differentiation factor 88 (MyD88)-dependent or MyD88-independent manner [6, 45]. NF-κB transcription factors are crucial for inflammation and important immune-regulatory genes [28, 46]. In regards to physiological conditions, NF-κB is segregated in the cytoplasm which is by binding to inhibitory proteins such as IκBs. However, stimuli trigger the formation of IKK complexes comprising IKKα, IKKβ, and NF-κB essential modulator which is also known as IKKγ [27]. The activated cells result in the phosphorylation and proteasome-mediated diminish in the level of IκB [47]. The sequence free NF-κB translocates into the nucleus, links to its accord sequence, and activates the target genes to do transcription [26]. The results of protein expression and immunofluorescence staining assays showed an increase in NF-κB p65 translocation into the nucleus (Fig. 3a, d). ACE inhibited the NF-κB-mediated translocation of p65 and phosphorylation of IκB and IKK α/β in a dose-dependent manner (Fig. 3b, c). These scrutinies suggest that ACE suppressed the initiation in the intracellular signaling cascades inhibiting the phosphorylation of IKK α/β. Consequently, it had suppressive effect on the output of pro-inflammatory mediators through blocking NF-κB-mediated p65 translocation (Fig. 5).

## Conclusion

In conclusion, this study demonstrated the anti-inflammatory effect of ACE in LPS-stimulated macrophages. ACE suppressed the yield of pro-inflammatory cytokines mediators in murine macrophage cell line, RAW 264.7 cells. It conducted via the regulation on the NF-κB signaling pathway, especially through the translocation of p65 and the activation of IκB. To confirm the effect of ACE on the output of inflammatory mediators for instance NO and PGE_2_, we evaluated their production in vitro as well as ex vivo with primary peritoneal macrophages. The findings indicate the effectiveness of ACE on LPS-induced inflammatory response in macrophages.

## Data Availability

The datasets used and/or analyzed during the current study are available from the corresponding author on reasonable request.

## Abbreviations

ACE: *A.chinensis* ethanol extract
TG: Lipopolysaccharidethioglycollate
NF-κB: Nuclear factor κB
NO: Nitric oxide
PGE_2_: Prostaglandin E2
iNOS: Inducible nitric oxide synthase
COX-2: Cyclooxygenase-2
(IL)-6: Interleukin
TNF-α: tumor necrosis factor-alpha
IKK: IκB kinase
IκB: Inhibitor of κB
